# Towards an inclusive digital health ecosystem

**DOI:** 10.2471/BLT.24.292020

**Published:** 2024-12-03

**Authors:** Yao Xie, Kayode Philip Fadahunsi, Carol Kelleher, Derjung M Tarn, Audrey Grace, John O' Donoghue

**Affiliations:** a Academic General Practice, School of Medicine, University College Dublin, C110 Health Sciences Centre, Belfield, Dublin 4, Ireland.; b Department of Primary Care and Public Health, Imperial College London, England.; c Department of Management and Marketing, University College Cork, Ireland.; d David Geffen School of Medicine, University of California Los Angeles, Los Angeles, United States of America.; e Business Information System, University College Cork, Ireland.

The United Nations (UN) defines digital inclusion as equitable, meaningful and safe access to the use, leadership and design of digital technologies, services and associated opportunities for all, regardless of location.^1^ Despite globalization and technological advancements, inequitable distribution of digital health benefits remains a critical challenge.^2^^–^^7^ Socioeconomically disadvantaged populations continue to be underserved,^7^ because ensuring that no one is left behind is difficult when relying solely on technological advancements to cover the most vulnerable, who can least afford digital technologies.^2^^,^^3^ Inclusive digital health should be a must,^2^ but efforts should go beyond technology.

Important barriers to achieving digital inclusion persist, especially within complex and diverse health-care environments. For example, at the microlevel, patients may struggle with limited access to affordable digital devices or reliable internet connection. Health workers, particularly in underfunded settings, may lack the necessary digital literacy to fully utilize electronic health records or telemedicine platforms. On a broader, system-wide level, health-care institutions often face challenges in integrating new technologies into existing workflows due to outdated infrastructure or fragmented systems.^2^^,^^4^ The effectiveness of digital health interventions is influenced by the capabilities of their users^2^ (patients and health workers) and by the interconnected efforts of multisectoral stakeholders, including policy-makers, technology developers, education providers at all levels and others.^2^^,^^3^^,^^5^ Each stakeholder contributes to serving a diverse patient population. The decisions these stakeholders make create ripple effects that shape the real-time experiences of end-users, ultimately affecting patient health outcomes. 

The challenge of achieving inclusive digital health is dynamic because social and technical barriers are interconnected and constantly evolving. These barriers influence one another in ways that require multisectoral collaborations among and between stakeholders,^2^^,^^3^^,^^5^ ranging from end-users, such as patients and health workers who directly interact with the technology, to other stakeholders who are not directly involved in immediate technological use, such as developers, technical support teams, researchers and regulatory bodies. Designing solutions that accommodate the diverse needs of all populations demands that stakeholders adopt a sociotechnical lens^8^ that considers the interplay between social factors (such as culture, inclusivity and accessibility) and technical aspects (such as design, functionality and data and system integration) in every decision. Doing so involves creating an environment where digital tools and human capacities work together to produce equitable and inclusive outcomes. Just as cooperation between multisectoral stakeholders is essential,^2^^,^^3^^,^^5^ the potential for true digital health inclusion lies in the integration of multilevel approaches. Acknowledging the autonomy of individuals who may opt out of digital services, and providing adequate non-digital solutions to meet their needs is also crucial as part of true inclusive digital health.^2^

Universal design, a process that enables and empowers population groups by improving human performance, health and wellness, and social participation,^9^ offers a promising opportunity for inclusive digital health. Originally rooted in the disability movement of the early 20th century, universal design has gained recognition across various sectors, extending its application beyond industrial design.^9^ For instance, in the development of emerging technological innovations, designers can present content in various formats and accommodate different interaction styles such as touch, voice, gesture controls, keyboard shortcuts and navigation systems. While the application of universal design principles has considerably improved digital accessibility, it often falls short of achieving true inclusivity, as exemplified by online video-based consultations.^7^ This type of consultation often offers improved accessibility to people living in rural, remote and disadvantaged communities, but it does not fully resolve challenges relating to the capacities of the end-users or personal competencies, issues that in-person consultations also fail to resolve.^2^^,^^3^^,^^5^^–^^7^^,^^10^ Additional factors affecting the adoption of online consultations include the user-friendliness of the technology and the preparedness of the health-care system – from governance to execution, and from ongoing optimizations to the readiness of information and communication technology infrastructure such as high-speed broadband or access to digital devices.^6^^,^^7^ Online video-based consultations serve as a microcosm of broader digital health innovations, encompassing technologies such as virtual reality, augmented reality and artificial intelligence.^11^

One of the primary reasons for the limitation of universal design in practice is that many accessibility features are added as an afterthought rather than being integrated into the core design process. Additionally, designers and developers frequently lack a comprehensive understanding of the diverse spectrum of user abilities and needs, particularly in digital health. This gap in knowledge can lead to oversimplified or incomplete solutions that are constrained by current technological capabilities, ultimately resulting in interventions that do not fully meet the needs of all users. Implementing comprehensive universal design features can be complex and costly, requiring not only substantial funding and time but also specialized knowledge. Environmental, social and political factors at both the country and global levels can affect the prioritization of inclusive design in the development of digital health solutions.

Well-implemented universal design principles do offer a promising pathway to inclusive digital health. For example, universal design can be used to enable patients with limited digital literacy to initiate an online video-based consultation using a familiar technology such as a telephone, or visually impaired individuals to have a fully audio-controlled platform. Translating universal design principles into softer domains such as decision-making, policy formulation and cultural participation is relevant to advancing inclusive digital health. For example, the successful transfer of universal design principles to the concept of universal design for learning in education^12^ empowers educators to create flexible learning environments that accommodate the diverse needs and capabilities of all learners.^12^ The success of universal design for learning, despite the difficulties of balancing flexibility with maintaining academic standards, underscores the importance of deep understanding of human diversity and the possibilities of embedding these principles into the design of decisions and solutions that benefit everyone.

We propose universal design for decision-making as a concept that supports a multilevel approach involving inclusive participation; clarity and accessibility in communication; agility in individual proactivity; cross-disciplinary, multilevel collaboration; human-centred design, feedback and iteration; and scalable equity across different levels. Universal design for decision-making tackles multilevel challenges at all levels, engaging stakeholders (from individuals to governments) and covering organizational, technological, educational and policy-related needs. This multilevel approach enables stakeholders to make responsible, inclusive decisions such as strategy, policy, technology and health promotion, fostering a culture where inclusivity is natural and expected. Embedding accessibility within digital health systems ensures that equitable and inclusive decisions are the default, creating a foundation for inclusive health.

The World Health Organization (WHO) launched the Global initiative on digital health in early 2024 to promote greater integration of inclusive digital health solutions worldwide. Other relevant initiatives for inclusive digital health include: (i) the UN Secretary-General’s roadmap for digital cooperation;^3^ (ii) The age of digital interdependence: report of the UN Secretary-General’s high-level panel on digital cooperation;^5^ (iii) the Pan American Health Organization’s eight guiding principles for digital transformation of the health sector;^2^ (iv) the National Health Service (England) framework for action on digital inclusion in health care;^13^ and (v) Governing health futures 2030.^14^ These initiatives emphasize that interdisciplinary collaboration is not a choice, but a necessity. However, these collective efforts have faced challenges regarding effectiveness, cost and implementation sustainability,^2^^–^^5^ because the initiatives are often weakened by lack of alignment across stakeholders, including government bodies, private sector actors and civil society. Moreover, the high costs associated with scaling digital solutions in low-resource settings pose considerable barriers. Additionally, shifting political climates, funding constraints and the rapid pace of technological development challenge the long-term sustainability of these efforts. Through deliberate efforts involving thoughtful planning of actions, collaboration and training, stakeholders have gradually moved from raising awareness to concrete actions. Many of those efforts inherently reflect the aim of universal design for decision-making of fostering greater inclusivity and contributing to global digital cooperation.

To complement the collective efforts above,^1^^–^^6^ in this article we have synthesized seven key dimensions that we consider crucial for optimizing inclusive digital health. All the relevant multisectoral stakeholders should consider these seven dimensions for optimizing inclusive digital health using the universal design for decision-making approach (Table 1).

**Table 1:** Inclusive digital health using universal design for decision-making approach

Dimension, definition	Core principles	Decision-making cues
**Policy development**		
Creation of guidelines, regulations and frameworks to ensure equitable access, usability and effectiveness of digital health technologies for all, including individuals with disabilities and diverse requirements	Establish inclusive guidelines and frameworks	Bridging digital divide, human rights, global governance, equity, resource allocations, infrastructure advancement and flexibility
**Technology-end development**		
Developing digital health solutions with a strong focus on accessibility, inclusivity and usability that address the needs of diverse users in health care from the perspective of technology providers and developers	Design accessible, inclusive and ethical digital solutions	Inclusive innovation, integration of technology and health care, education, ethics and responsible technology
**Languages and communication**		
Ensuring that digital health technologies and services are accessible to individuals who speak different languages and may have communication challenges or unique language preferences	Ensure accessibility for diverse languages and approaches	Cooperation, trust, effectiveness, comprehension, efficacy, respect and loops for feedback
**Lifelong user capacity-building**		
Continuous development and enhancement of individuals’ knowledge, skills, capabilities and awareness to effectively use digital health technologies and services throughout the users’ life. Users refer to individuals who need access to these technologies	Enhance digital literacy, skills and knowledge	Capacity-building, resilience, empowerment, awareness to actions, human rights and education
**Inclusion of underserved groups in the digital health landscape**		
Groups in vulnerable situations, such as those in rural areas or with low digital literacy, who face significant barriers to accessing and using digital health technologies and services. These challenges arise from factors including motivation, material resources, skills, usage patterns and individual identities. Key issues include limited digital literacy, ageing populations, low socioeconomic status, privacy concerns and technological disparities	Prioritize engagement with marginalized populations, who are in vulnerable situations, rural areas, or individuals with low digital literacy	Vulnerable populations, social inclusion, access and human rights
**Secure, trustworthy digital health and information systems**		
Refers to robust privacy and data security measures, transparency and ensuring systems uphold human rights	Protect personal data and enhance trust	Data privacy, trust, transparency, sustainability, human rights and information infrastructure
**Cross-dimension collaboration**		
Involves multistakeholder cooperation that brings together diverse voices, including civil society, academia and the private sector. This collaboration aims to include perspectives from low-income countries and traditionally marginalized groups such as women, youth, indigenous communities, rural populations and older adults	Encourage cross-sector partnership, multilevel cooperation and diverse voices	Multistakeholder cooperation, multisectoral, multilevel stakeholder engagement, sustainability, public–private partnerships, interdisciplinary perspectives, resource allocations for equity and justice

As technological development accelerates, our capacity for rapid response, collaboration and adaptive governance becomes essential.^3^^,^^5^ The UN Secretary-General’s high-level panel on digital cooperation highlights the urgency of establishing cooperative models that uphold human rights and ensure digital equity.^3^^,^^5^ Integrating sociotechnical methods with universal design for decision-making provides a strategic framework to embed inclusivity in decision-making, supporting digital health solutions that meet the diverse needs of all populations in an agile and forward-looking way. The sociotechnical approach emphasizes the dynamic interplay between social and technical factors in system design; while universal design for decision-making fosters an inclusive ecosystem via multisectoral stakeholders, thus enabling more holistic and inclusive decision-making. Combining these methods offers a strategic direction for multisectoral stakeholders to guide the design process towards more inclusive outcomes. This integrated strategy maximizes the strengths of each method while mitigating limitations, ensuring the diverse needs of the global digital health community are met. 

Each decision, whether a policy development, a technological innovation or a strategic partnership, could be a building block for a more equitable, resilient and inclusive digital health ecosystem. When these decisions are grounded in principles of inclusivity, they culminate in transformative outcomes towards the goal of achieving equitable health access and universal health coverage. This goal is a shared responsibility among individuals, governments, civil society and health-care system stakeholders.^4^

Creating an inclusive digital health landscape is a continual, transformative process requiring numerous inclusive decisions informed by intergenerational, collective efforts.^4^

Achieving this goal demands an approach that integrates diverse concepts and methods such as sociotechnical considerations and universal design for decision-making. These consensus-driven approaches should serve as fundamental avenues for addressing both current and future challenges, evolving over time to become a core mechanism for navigating the dynamic and fast-changing health-care landscape. Establishing such a robust, dynamic system necessitates variety, testing and a collective intelligence at local, regional and global scales.
